# Comparison between Atasoy-Kleinert V-Y Advancement Flap and Figueiredo Techniques for the Treatment of Transverse and Dorsal Oblique Fingertip Injuries

**DOI:** 10.1055/s-0042-1749203

**Published:** 2022-06-02

**Authors:** Leandro Azevedo de Figueiredo, Rafael de Souza Ribeiro, Pedro Hemerly Figueiredo, André Luiz Machado Lima, Felipe Mantovani de Oliveira, Dalton Silva de Oliveira Júnior

**Affiliations:** 1 Vitória Apart Hospital, Serra, ES, Brasil; 2 Hospital Jayme dos Santos Neves, Serra, ES, Brasil; 3 Santa Casa de Misericórdia de Vitória, Vitória, ES, Brasil

**Keywords:** finger injuries, microsurgery, surgical flaps

## Abstract

**Objective** Compare the results of the treatment of transverse and dorsal oblique fingertip lesions with Atasoy-Kleinert V-Y advancement flap or Figueiredo Technique (FT).

**Method** A total of 21 patients who suffered acute trauma in any finger with consequent transverse or oblique fingertip lesions were selected in a public referral hospital in high complexity trauma. Of these, 10 patients were treated with the Atasoy-Kleinert V-Y advancement flap technique and 11 with the FT. The aesthetic and functional results were compared based on four criteria: evolution of infectious process during treatment; capacity for static and dynamic discrimination between two points; neuroma formation; and aesthetic and functional evaluation of nail growth.

**Results** None of the studied groups developed neuroma or infectious process during treatment. In the general clinical evaluation, it was observed that no variable presented a statistically significant association, that is, both groups presented similar behaviors in the postoperative period with *p* value > 0.05 when comparing all the included variables.

**Conclusion** The present study concluded that the FT is as efficient as the Atasoy-Kleinert V-Y advancement flap technique, both of which can be used for the treatment of transverse and dorsal oblique fingertip injuries, two techniques with excellent results for such lesions.

## Introduction

Fingertip injuries are defined as lesions in the distal portion of the fingers, where the flexor and extensor tendons are. They are often found by hand surgeons in the urgency and emergency setting and represent the most common type of upper limb amputation; furthermore, they are constantly associated with concomitant nail bed lesions. 1 2

Despite its high prevalence, treatment is often not properly conducted in the patient's first care, thus causing chronic deformities and dysfunctions of the nail and fingertip over time. The lack of professionals prepared for the management of this type of injury in most emergency care in Brazil contributes significantly to these poor outcomes. 3 4

To choose the surgical treatment method, it is important to consider individual variables of each patient and lesion, such as age, occupation, number of injured fingers, bone exposure, time of injury, and viability of digit reconstruction. 5 As well as variables inherent to the surgical method, such as: cost, complexity of the reproduction of the technique and possible secondary damage generated to the patient. Finally, given the range of treatment options, doctors should choose the one that promotes greater comfort, better recovery, and greater results. 3 6

The aim of this study is to compare Figueiredo's surgical technique (FT) 3 with the Atasoy-Kleinert V-Y advancement flap technique, 7 evaluating the functional and aesthetic results for the treatment of transverse and dorsal oblique fingertip lesions.

## Materials and Methods

The study protocol was approved by the Ethics Committee on Research Involving Human Beings of our institution. This is a prospective, randomized by lot, without masking of patients, convenience sample clinical trial. 8 9

From July 2018 to December 2018, 21 patients, 17 males and 4 females, treated in the emergency room of a public hospital with reference in high complexity trauma, were selected for the study. The inclusion criterion was acute trauma to any finger with consequent transverse or dorsal oblique fingertip injury. Volar oblique lesions were excluded from the study, as it is not recommended to treat them with the Atasoy-Kleinert V-Y advancement flap technique. 7 Therefore, only lesions that can be treated by both methods will be under analysis.

**Fig. 1:**
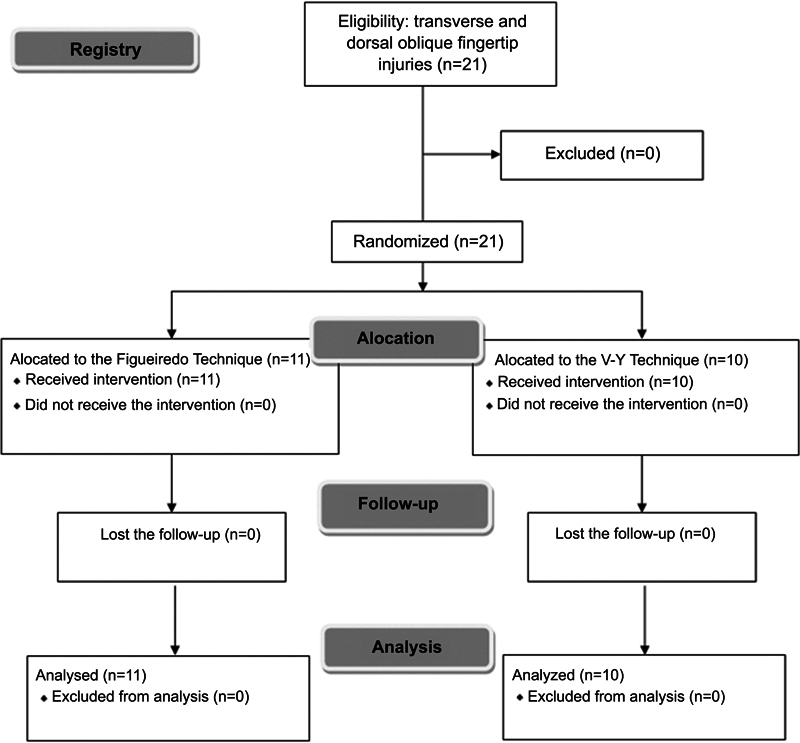
Consolidated Standards Flowchart of Test Reports (CONSORT).

The patients were numbered according to their order of care, the first being number 1, the second being number 2, and so on. Then, randomization was performed with the online mechanism Sealed Envelope (Sealed Envelope LTD. London, UK), and they were divided into two groups: A (patients 2, 4, 6, 8, 10, 11, 15, 16, 18, and 20) and B (patients 1, 3, 5, 7, 9, 12, 13, 14, 17, 19, and 21). We also used the Consolidated Standards Flowchart of Test Reports (CONSORT) represented in Fig. 1 .

**Fig. 2:**
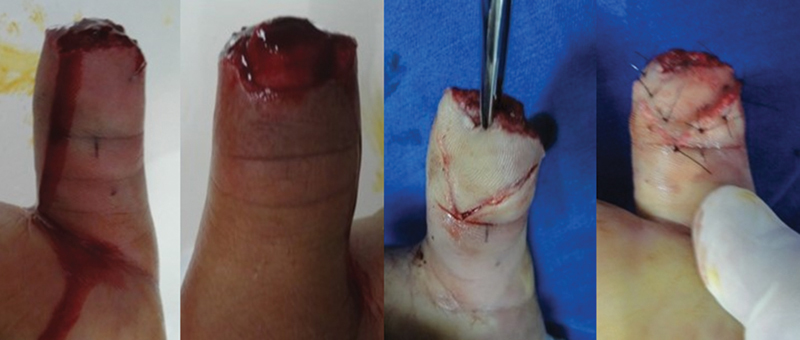
Initial injury and surgical procedure – V-Y technique.

Group A was submitted to treatment with the Atasoy-Kleinert V-Y advancement flap technique, described in the literature as an option for the treatment of transverse or dorsal oblique injuries, already used for decades by surgeons around the world, 10 11 12 following the technical procedures described in their original work 7 ( Fig. 2 ).

**Fig. 3:**
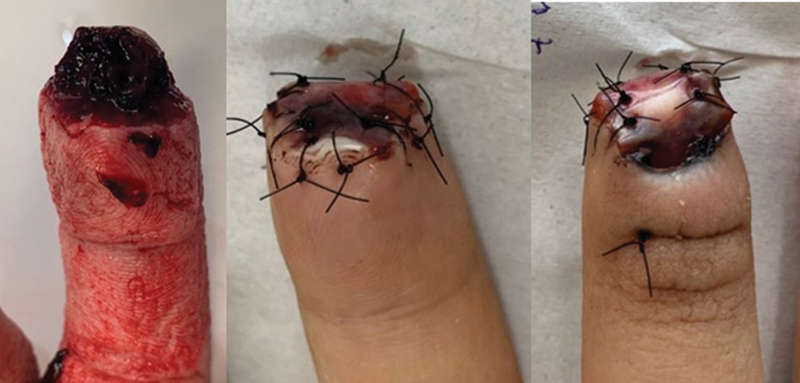
Initial injury and surgical procedure – Figueiredo technique.

**Fig. 4:**
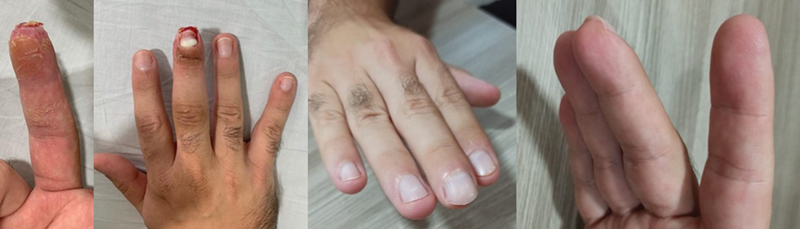
Respectively, result after 6 weeks and 3 months of the application of FT.

Group B was submitted to treatment with the FT, which consists of promoting wound protection through a polypropylene prosthesis that is cut out of sterile vials of saline, in the wound's exact shape, sutured on its healthy edges through simple stitches, and accommodated without pressing the bloody area. In traumas in which there were concomitant lesions of the nail bed, this was previously sutured with nonabsorbable threads type prolene 7.0 or 8.0 and then protected by the prosthesis, which in this case was first fixed under the eponyquium with U-point and then sutured on the edges of the lesion 3 ( Figs. 3 and 4 ).

All procedures were performed by the same surgical team, composed of Orthopedists and Hand Surgeons and Microsurgeons, members of the Brazilian Society of Hand Surgery (SBCM). The surgery was timed from the confirmation of lidocaine block to the last suture point.

After completion of the surgery, a dressing was made with sterile gauze and micropore. The first change occurred after 7 days, in the first postoperative return consultation. All were followed weekly by the hand surgery and microsurgery team of the hospital where the study was conducted, until their full recovery. In patients treated by the V-Y technique, the stitches were removed after two weeks, while in patients undergoing treatment with FT, the prosthesis was removed six weeks after placement.

In the third postoperative month, the patients were evaluated by the same team based on 4 criteria. The first was considering the evolution of infectious process during treatment, according to the records of medical records of outpatient consultations, being classified as “present” or “absent”. If present, culture and antibiogram analysis would be carried out.

The second criterion evaluated the capacity of static and dynamic discrimination between two points, through the Weber test, 13 comparing with the non-traumatized contralateral side. In this item, the patient was classified as “normal” skilled case to discriminate two points with distance less than 6 mm; “satisfactory” between 6 and 10 mm; “poor” between 11 and 15 mm; “protective” when distinguishes only one point, and “anesthesia” if it can't distinguish any point.

The third criterion evaluated the formation of neuroma by the percussion test on the wound, classifying it as “present” or “absent”.

The fourth criterion was the aesthetic and functional evaluation of nail growth, subdivided into three items, as described by Silva and Gerhardt, 14 also compared to the contralateral side. Item 1: Nail growth, rated 0 when there was no growth; 1, when there was partial growth with supports, and 2 with normal growth. Item 2: Nail size, classified with 0, if less than or equal to 50% on the opposite side, 1 when between 50 and 75%, and 2 if greater than or equal to 75%. Item 3: Nail shape, classified by 0 when there was a significant deformity in the vertical plane, 1 when a small deformity was observed, and 2 without deformities. These results were then added and classified as “bad” when the sum has a value less than three, “regular” when three or four, and “good” when five or six. Other variables are presented in Table 1 .

**Table 1:** Variables of characterization of the lesion and its mechanism of trauma and patient occupation

Variables		N	N%
Profession	Mechanic	6	26.6%
	Janitorial services	5	23.8%
	Stay-at-home person	2	9.5%
	Bricklayer	2	9.5%
	Carpenter	1	4.8%
	Electrician	1	4.8%
	Delivery person	1	4.8%
	Painter	1	4.8%
	Accountant	1	4.8%
	Salesperson	1	4.8%
Injured side	R	6	26.6%
	L	15	71.4%
Injured finger	1	5	23.8%
	2	8	38.1%
	3	5	23.8%
	4	1	4.8%
	5	2	9.5%
Mechanism	Explosive	1	4.8%
	Automatic door	1	4.8%
	Hydraulic press	1	4.8%
	Belt	2	9.5%
	Crushing	4	19.0%
	Bladed weapon	5	23.8%
	Toothed saw	7	33.3%

Due to their qualitative nature, the data were analyzed by frequencies and percentages.

The association of categorical variables was performed by means of the Chi-square test, and for expected frequencies lower than 5, the Fisher exact test was performed in the case of crosstables that presented the matrix shape 2 × 2.

For the associations, a significance level of 5% was considered, so *p* -values lower than 0.05 indicated a significant result.

The data were organized in an Excel (Google LLC. Mountain View, CA, US) spreadsheet and analyzed in the Statistical Package for the Social Sciences (SPSS, IBM Corp. Armonk, NY, US) version 27 software.

## Results

None of the groups of patients presented infectious or neuroma during the study and there was no significant difference in surgical time for the techniques employed.

In the general clinical evaluation, it was observed that no variable presented a statistically significant association, i.e., both groups presented similar behaviors in the postoperative period. The results are presented in Table 2 .

**Table 2:** Evaluation results three months after surgery

Variables		Figueiredo		V-Y		*P-* value
		N	N%	N	N%	
Infection	No	11	100,0%	10	100,0%	
Two static points	Normal	6	54,5%	4	40,0%	0,67
	Satisfactory	5	45,5%	6	60,0%	
Two dynamic points	Normal	8	72,7%	4	40,0%	0,198
	Satisfactory	3	27,3%	6	60,0%	
Neuroma	No	11	100,0%	10	100,0%	
Nail growth	1	1	9,1%	0	0,0%	0,999
	2	10	90,9%	10	100,0%	
Nail size	0	2	18,2%	0	0,0%	0,261
	1	5	45,5%	3	30,0%	
	2	4	36,4%	7	70,0%	
Nail shape	0	2	18,2%	0	0,0%	0,473
	1	6	54,5%	5	50,0%	
	2	3	27,3%	5	50,0%	
Nail complete	2	2	18,2%	0	0,0%	0,134
	4	3	27,3%	3	30,0%	
	5	5	45,5%	2	20,0%	
	6	1	9,1%	5	50,0%	

## Discussion

The V-Y advancement flap surgery described by Atasoy and Kleinert is already widely used by surgeons. The triangular flap is often used in fingertip amputation reconstructions with bone exposure and is indicated for transverse or oblique back lesions. This procedure was not recommended for volar oblique lesion. Additionally, it is necessary to use a donor area of cutaneous tissue, coming from a nontraumatized region from the injured finger. 5 15

The FT, in turn, besides presenting a broader indication than the established Atasoy technique, because it also applies to the treatment of volar oblique lesions, makes it unnecessary to use a healthy donor area, avoiding secondary damage to the patient. 3

Both techniques are easy to reproduce and can be carried out with low-cost materials. 3 15 The material used as a prosthesis in FT should be sufficiently resistant to protect the site of injury against painful stimuli and external forces, until proper healing occurs, but should not be so rigid as to cause tissue deformities. Thus, the material that proved most appropriate was the saline bag composed of polypropylene, because it was sterile, low cost and easily accessible in surgical centers throughout the country. 3

In his initial description, Atasoy highlights the sensitivity at the fingertip as an advantage of performing the triangular flap. 7 15 As presented in this study, coverage through FT presents results similar to the technique already established.

In the evaluation of the capacity of static discrimination between two points, 54.5% of the patients in the FT group were classified as normal and 45.5% as satisfactory. In the V-Y flap group, 40% of patients were classified as normal and 60% as satisfactory.

Regarding the capacity of dynamic discrimination between two points, the group submitted to reconstruction by V-Y maintained the results presented in the static evaluation. While in the TF group, there was an improvement in the results, with 72.7% classified as normal and 27.3% as satisfactory.

Considering the nail growth item in the FT group, 18.2% of the patients presented poor results, 27.3% regular, and 54.6% good results. In the V-Y group, no patient had poor results, 30% had regular, and 70% good.

A common concern in cases where there is bone exposure is the possibility of infection. 16 In none of the studied groups, this complication was present, evidencing that both techniques are safe in this item. Although it is a concern when flaps are performed, in this study there were no cases of digital skin necrosis in patients operated with the V-Y technique.

It is important to note that, in patients undergoing FT, there is the most exuberant formation of fibrin tissue between the second and third weeks, which has typical yellowish coloration and can lead the patient to imagine that it is purulent secretion. For this reason, patients should be alerted in advance about this important transitional stage of treatment. This yellowish tissue is precisely the ideal mold and will be gradually replaced by granulation tissue that will then undergo epithelialization process until complete healing. During this period, there is continuous improvement in the aspect of the entire area around the wound, with decreased edema and absence of phlogistic signs. 3

The established V-Y technique is effective and presents excellent results when performed by a specialized professional. The FT showed the same efficacy and emerges as an alternative for the treatment of fingertip lesions, also standing out due to its easy execution—which can be performed by a larger number of surgeons—and for providing protection to the site of injury without requiring a healthy donor area, thus allowing wound healing to occur by a second intention.

As a limitation of the study, the non-correlation between the severity of the initial lesion and the outcome after treatment can be highlighted. More severe lesions with greater soft tissue involvement, associated with nail bed injury and fractures, generally present worse aesthetic and functional results and with greater sequelae, regardless of the surgical technique applied.

Another limitation of the study was the small sample, which occurred due to the demand for cases available in the proposed period for the study. These results can be used for future studies of hand surgery techniques.

## Conclusion

The present study concluded that the FT is as efficient as the Atasoy-Kleinert V-Y advancement flap technique, both of which can be used for the treatment of transverse and dorsal oblique fingertip lesions, presenting good aesthetic and functional results for such lesions.
