# Supplementary material for: Comparison between Atasoy-Kleinert V-Y Advancement Flap and Figueiredo Techniques for the Treatment of Transverse and Dorsal Oblique Fingertip Injuries
Source: Rev Bras Ortop (Sao Paulo). 2022 Jun 2;59(5):e712–8. doi: 10.1055/s-0042-1749203 (PMC11624923; doi:10.1055/s-0042-1749203)
Supplement: Related texts

# Research article: Comparação entre as técnicas de Figueiredo e de reconstrução volar V-Y de Atasoy-Kleinert no tratamento de lesões transversas e oblíquas dorsais de ponta de dedo

**Keywords:** lesões dos dedos, microcirurgia, retalhos cirúrgicos

## Abstract

**Objetivo** Comparar os resultados do tratamento de lesões transversais e oblíquas dorsais de ponta de dedo, tratadas pelas técnicas de reconstrução volar VY de Atasoy-Kleinert e a de Figueiredo.

**Método** Foram selecionados em um hospital público de referência em trauma de alta complexidade 21 pacientes que sofreram trauma agudo em qualquer dedo da mão com consequentes lesões transversas ou oblíquas dorsais de ponta de dedo. Destes, 10 pacientes foram tratados pela técnica de reconstrução volar V-Y de Atasoy-Kleinert e 11 pela Técnica de Figueiredo (TF) e os resultados estéticos e funcionais foram comparados com base em quatro critérios: evolução de processo infeccioso durante o tratamento; capacidade de discriminação estática e dinâmica entre dois pontos; formação de neuroma; e avaliação estética e funcional do crescimento da unha.

**Resultados** Nenhum dos grupos estudados desenvolveu neuroma ou processo infeccioso durante o tratamento. Na avaliação clínica geral observou-se que nenhuma variável apresentou associação estatisticamente significativa, ou seja, ambos grupos apresentaram comportamentos semelhantes no pós-operatório com um valor-p > 0,05 na comparação de todos os critérios estudados.

**Conclusão** O presente estudo concluiu que a Técnica de Figueiredo é tão eficiente quanto a técnica de reconstrução volar V-Y de Atasoy-Kleinert, ambas podem ser utilizadas para o tratamento de lesões transversas e oblíquas dorsais em ponta de dedos, sendo duas técnicas de excelentes resultados para tais lesões.

## Introdução

Lesões de ponta de dedo são definidas como as lesões na porção distal dos dedos, onde os tendões flexores e extensores estão. Elas são frequentemente encontradas pelos cirurgiões de mão no cenário de urgência e emergência, e representam o tipo mais comum de amputação em membro superior; além disso, estão constantemente associadas a lesões concomitantes de leito ungueal. 1 2

Apesar de sua alta prevalência, muitas vezes, o tratamento não é conduzido adequadamente no primeiro atendimento do paciente, causando assim, deformidades e disfunções crônicas da unha e da ponta do dedo ao longo do tempo. A falta de profissionais preparados para o manejo desse tipo de lesão na maioria dos pronto-atendimentos do Brasil contribui significativamente para esses desfechos negativos. 3 4

Para escolha do método de tratamento cirúrgico é importante que se leve em conta variáveis individuais de cada paciente e de sua lesão, como: idade, ocupação, número de dedos lesados, exposição óssea, tempo de lesão e viabilidade de reconstrução do dígito. 5 Assim como variáveis inerentes ao método cirúrgico, como, por exemplo: custo, complexidade da reprodução da técnica e possíveis danos secundários gerados ao paciente. Por fim, diante do leque de opções de tratamento, o médico deve escolher aquele que promova maior conforto, melhor recuperação e ótimo resultado. 3 6

O objetivo deste estudo é comparar a técnica cirúrgica de Figueiredo (TF) 3 com a técnica de reconstrução volar V-Y de Atasoy-Kleinert, 7 avaliando os resultados funcionais e estéticos para o tratamento de lesões transversas e oblíquas dorsais da ponta de dedo.

## Materiais e Métodos

O protocolo de estudo foi aprovado pelo Comitê de Ética em Pesquisa Envolvendo Seres Humanos da nossa instituição. Trata-se de um ensaio clínico, prospectivo, randomizado por sorteio, sem mascaramento dos pacientes, de amostra por conveniência. 8 9

No período de Julho de 2018 até Dezembro de 2018, 21 pacientes, 17 do gênero masculino e 4 do feminino, atendidos no pronto atendimento de um hospital público de referência em trauma de alta complexidade, foram selecionados para o trabalho. O critério de inclusão foi trauma agudo em qualquer dedo da mão com consequente lesão transversa ou oblíquas dorsais de ponta de dedo. Lesões oblíquas volares foram excluídas do trabalho, pois não é recomendado seu tratamento por meio da técnica de reconstrução volar V-Y de Atasoy-Kleinert. 7 Com isso, apenas lesões que podem ser tratadas por ambos os métodos de estudos estarão em análise.

Os pacientes foram numerados de acordo com sua ordem de atendimento, sendo o primeiro o número 1, o segundo o número 2 e assim por diante. Em seguida, procedeu-se à randomização, realizada com o mecanismo online Sealed Envelope (Sealed Envelope LTD. Londres, Reino Unido), separando-os em dois grupos: A (pacientes 2, 4, 6, 8, 10, 11, 15, 16, 18 e 20 e B (pacientes 1, 3, 5, 7, 9, 12, 13, 14, 17, 19 e 21). Fluxograma de Normas Consolidadas de Relatórios de Ensaio (CONSORT) representado na Fig. 1 .

**Fig. 1:** Fluxograma de Normas Consolidadas de Relatórios de Ensaio (CONSORT).

O grupo A foi submetido ao tratamento pela técnica de retalho volar V-Y de Atasoy *,* descrita na literatura como uma opção para o tratamento de lesões transversas ou oblíquas dorsais, já utilizada há décadas por cirurgiões de todo o mundo, 10 11 12 seguindo os procedimentos técnicos descritos em seu trabalho original 7 ( Fig. 2 ).

**Fig. 2:** Lesão inicial e procedimento cirúrgico – Técnica de VY.

O grupo B foi submetido ao tratamento pela TF, que consiste em promover proteção a ferida através de uma prótese de polipropileno que é recortada de frascos estéreis de soro fisiológico no formato exato da mesma, suturada em suas bordas sadias através de pontos simples e acomodada sem pressionar a área cruenta. Nos traumas em que existiam lesões concomitantes do leito ungueal, esse foi previamente suturado com fios inabsorvíveis tipo prolene 7.0 ou 8.0 e em seguida protegido pela prótese, que nesse caso era primeiramente fixada sob o eponíquio por ponto em U e em seguida suturada nas bordas da lesão 3 ( Figs. 3 e 4 ).

**Fig. 3:** Lesão inicial e procedimento cirúrgico – Técnica de Figueiredo.

**Fig. 4:** Respectivamente, resultado após 6 semanas e 3 meses da aplicação da TF.

Todos os procedimentos foram realizados pela mesma equipe cirúrgica, composta por Ortopedistas e Cirurgiões de Mão e Microcirurgia, membros da Sociedade Brasileira de Cirurgia da Mão (SBCM). O tempo de cirurgia foi cronometrado a partir da confirmação do bloqueio com lidocaína até o último ponto de sutura.

Após finalização da cirurgia, foi feito curativo com gaze estéril e micropore. A primeira troca ocorreu após 7 dias, na primeira consulta de retorno pós-operatório. Todos foram acompanhados semanalmente, pela equipe de cirurgia da mão e microcirurgia do hospital onde o estudo foi realizado, até a sua total recuperação. Nos pacientes tratados pela técnica de VY, os pontos foram retirados após duas semanas, enquanto nos pacientes submetidos ao tratamento com a TF, a prótese foi retirada seis semanas após a sua colocação.

No terceiro mês de pós-operatório, os pacientes foram avaliados pela mesma equipe com base em 4 critérios. O primeiro sobre a evolução de processo infeccioso durante o tratamento, de acordo com os registros de prontuários das consultas ambulatoriais, sendo classificados como “presente” ou “ausente”. Caso presente se procederia a análise de cultura e antibiograma.

O segundo critério avaliou a capacidade de discriminação estática e dinâmica entre dois pontos, através do teste de Weber, 13 comparando com o lado contralateral não traumatizado. Neste quesito, o paciente era classificado como “normal” caso hábil a discriminar dois pontos com distância menor que 6mm; “satisfatório” entre 6 e 10 mm; “pobre” entre 11 e 15 mm; “protetora” quando distingue apenas um ponto, e “anestesia” se não consegue distinguir nenhum ponto.

O terceiro critério avaliou, pelo teste da percussão sobre a ferida, a formação de neuroma, classificando como “presente” ou “ausente”.

O quarto critério foi a avaliação estética e funcional do crescimento da unha, subdividido em três quesitos, conforme descrito por Silva e Gerhardt, 14 também comparado ao lado contralateral. Quesito 1: Crescimento da unha, classificado como 0 quando não havia crescimento; 1, quando havia crescimento parcial com apoios e 2 com crescimento normal. Quesito 2: O tamanho da unha, classificados com 0, se menor ou igual a 50% do lado oposto, 1 quando entre 50 e 75% e 2 se maior ou igual a 75%. Quesito 3: A forma da unha, classificado por 0 quando havia uma deformidade significativa no plano vertical, 1 quando uma pequena deformidade era observada e 2 sem deformidades. Esses resultados eram então somados e classificados como “ruins” quando a soma apresenta valor menor que três, “regular” quando três ou quatro e “bom” quando cinco ou seis. Outras variáveis estão apresentadas na Tabela 1 .

**Tabela 1:** Variáveis de caracterização da lesão e seu mecanismo de trauma e ocupação do paciente

Variáveis		N	N°
Profissão	Mecânico	6	26,6%
	Aux. serviços gerais	5	23,8%
	Do lar	2	9,5%
	Pedreiro	2	9,5%
	Carpinteiro	1	4,8%
	Eletricista	1	4,8%
	Entregador	1	4,8%
	Pintor	1	4,8%
	Técnico contábil	1	4,8%
	Vendedor	1	4,8%
Lado lesado	D	6	26,6%
	E	15	71,4%
Dedo lesado	1	5	23,8%
	2	8	38,1%
	3	5	23,8%
	4	1	4,8%
	5	2	9,5%
Mecanismo	Explosivo	1	4,8%
	Porta automática	1	4,8%
	Prensa hidráulica	1	4,8%
	Correia	2	9,5%
	Esmagamento	4	19,0%
	Arma branca	5	23,8%
	Serra denteada	7	33,3%

Devido a sua natureza qualitativa, os dados foram analisados por frequências e percentuais.

A associação de variáveis categóricas foi realizada por meio do teste Qui-quadrado, sendo que para frequências esperadas menores do que 5, foi realizado o teste exato de Fisher no caso de tabelas cruzadas que apresentarem a forma de matriz 2 × 2.

Para as associações foi considerado um nível de significância de 5%, assim valores de *p* menores do que 0,05 indicam resultado significativo.

Os dados foram organizados em planilha Excel (Google LLC. Mountain View, Califórnia, EUA) e analisados no programa Statistical Package for the Social Sciences (SPSS, IBM Corp. Armonk, Nova Iorque, EUA) versão 27.

## Resultados

Nenhum dos grupos de pacientes apresentou quadro infeccioso ou neuroma durante o estudo e não houve diferença significativa no tempo cirúrgico para as técnicas empregadas.

Na avaliação clínica geral observou-se que nenhuma variável apresentou associação estatisticamente significativa, ou seja, ambos os grupos apresentaram comportamentos semelhantes no pós-operatório. Os resultados estão apresentados na Tabela 2 .

**Tabela 2:** Resultados da avaliação três meses após cirurgia

Variáveis		Figueiredo		V-Y		Valor de *p*
		N	N%	N	N%	
Infecção	Não	11	100,0%	10	100,0%	
Dois pontos estático	Normal	6	54,5%	4	40,0%	0,67
	Satisfatório	5	45,5%	6	60,0%	
Dois pontos dinâmico	Normal	8	72,7%	4	40,0%	0,198
	Satisfatório	3	27,3%	6	60,0%	
Neuroma	Não	11	100,0%	10	100,0%	
Unha crescimento	1	1	9,1%	0	0,0%	0,999
	2	10	90,9%	10	100,0%	
Unha tamanho	0	2	18,2%	0	0,0%	0,261
	1	5	45,5%	3	30,0%	
	2	4	36,4%	7	70,0%	
Forma da unha	0	2	18,2%	0	0,0%	0,473
	1	6	54,5%	5	50,0%	
	2	3	27,3%	5	50,0%	
Unha total	2	2	18,2%	0	0,0%	0,134
	4	3	27,3%	3	30,0%	
	5	5	45,5%	2	20,0%	
	6	1	9,1%	5	50,0%	

## Discussão

A cirurgia de retalho de avanço volar V-Y descrita por Atasoy e Kleinert já é amplamente utilizada por cirurgiões. O flap triangular é frequentemente usado nas reconstruções de amputação de ponta de dedo com exposição óssea, sendo indicado para lesões transversais ou oblíquas dorsais. Este procedimento não é recomendado para lesão oblíqua volar. Além disso, faz-se necessário a utilização de uma área doadora de tecido cutâneo, advinda de uma região não traumatizada do dedo lesionado. 5 15

A TF, por sua vez, além de apresentar indicação mais ampla que a estabelecida técnica de Atasoy, pois aplica-se também para o tratamento de lesões oblíquas volares, torna desnecessária a utilização de uma área doadora sadia, evitando um dano secundário ao paciente. 3

Ambas as técnicas são de fácil reprodutibilidade e realizadas com materiais de baixo custo. 3 15 O material utilizado como prótese na TF deve ser suficientemente resistente para proteger o local da lesão contra estímulos dolorosos e forças externas, até que ocorra a cicatrização adequada, porém não deve ser tão rígido a ponto de provocar deformidades teciduais. Sendo assim, o material que se mostrou mais adequado foi a bolsa de soro fisiológico composta de polipropileno, por ser estéril, de baixo custo e facilmente acessível nos centros cirúrgicos de todo o país. 3

Na sua descrição inicial, Atasoy destaca a sensibilidade na ponta do dedo como vantagem da realização do retalho com flap triangular. 7 15 Como apresentado neste estudo, a cobertura por meio da TF apresenta resultados semelhantes à técnica já consagrada.

Na avaliação da capacidade de discriminação estática entre dois pontos, 54,5% dos pacientes do grupo da TF foram classificados como normal e 45,5% como satisfatório. Já no grupo do retalho V-Y, 40% dos pacientes foram classificados como normal e 60% como satisfatório.

Quanto a capacidade de discriminação dinâmica entre dois pontos, o grupo submetido a reconstrução por V-Y, manteve os resultados apresentados na avaliação estática. Enquanto no grupo da TF houve uma melhora dos resultados, com 72,7% classificados como normal e 27,3% como satisfatório.

No quesito crescimento da unha, no grupo da TF, 18,2% dos pacientes apresentaram resultado ruim, 27,3% resultado regular e 54,6% resultado bom. No grupo do V-Y, nenhum paciente foi classificado como ruim, 30% como regular e 70% como bom.

Uma preocupação comum em casos com exposição óssea é a possibilidade de infecção. 16 Em nenhum dos grupos estudados essa complicação se fez presente, evidenciando que ambas as técnicas se mostram seguras nesse quesito. Embora seja uma preocupação quando são realizados retalhos, neste estudo não houve casos de necrose cutânea digital nos pacientes operados com a técnica de V-Y.

É importante observar que, nos pacientes submetidos a TF, ocorre a formação mais exuberante de tecido fibrinoso entre a segunda e terceira semanas, que tem coloração amarelada típica e pode levar o paciente a imaginar tratar-se de secreção purulenta. Por esse motivo os pacientes devem ser alertados previamente sobre essa importante etapa transitória do tratamento. Esse tecido amarelado é justamente o molde ideal e será gradativamente substituído por tecido de granulação que, em seguida, passará por processo de epitelização até a completa cicatrização. Durante esse período, observa-se melhoria contínua do aspecto de toda área ao redor da ferida, com diminuição do edema e ausência de sinais flogísticos. 3

A consagrada técnica de V-Y é eficaz e apresenta ótimos resultados quando realizada por um profissional especializado. A TF mostrou a mesma eficácia e surge como uma alternativa para o tratamento de lesões em ponta de dedo, destacando-se ainda por sua fácil execução – podendo ser realizada por um maior número de cirurgiões – e por conferir proteção ao sítio de lesão sem necessitar de área doadora saudável, permitindo, assim, que ocorra a cicatrização da ferida por segunda intenção.

Como limitação do estudo, pode-se destacar a falta de correlação entre a gravidade da lesão inicial e o desfecho final após o tratamento. Lesões mais graves com maior acometimento de partes moles, associadas a lesão de leito ungueal e fraturas, em geral, apresentam resultado estético e funcional piores e com maiores sequelas, independente da técnica cirúrgica aplicada.

Outra limitação do estudo foi quanto à pequena amostra utilizada, o que ocorreu devido à demanda de casos disponíveis no período proposto para a realização do estudo. Esses resultados podem ser utilizados para futuros estudos de técnicas de cirurgia da mão.

## Conclusão

O presente estudo concluiu que a TF é tão eficiente quanto a técnica de reconstrução volar V-Y de Atasoy-Kleinert, ambas podendo ser utilizadas para o tratamento de lesões transversas e oblíquas dorsais em ponta de dedos, apresentando bons resultados estéticos e funcionais para tais lesões.
